# Prevalence of persistent hypertension following pregnancy complicated by hypertensive disorders in low- and middle-income countries: a systematic review

**DOI:** 10.3389/fgwh.2024.1315763

**Published:** 2024-03-01

**Authors:** Moses Mukosha, Abigail Hatcher, Wilbroad Mutale, Mwansa Ketty Lubeya, Jamie L. Conklin, Benjamin H. Chi

**Affiliations:** ^1^ Department of Pharmacy, School of Health Sciences, University of Zambia, Lusaka, Zambia; ^2^ School of Public Health, Faculty of Health Sciences, University of the Witwatersrand, Johannesburg, South Africa; ^3^ Department of Health Behavior, University of North Carolina at Chapel Hill, Chapel Hill, NC, United States; ^4^ School of Public Health, University of Zambia, Lusaka, Zambia; ^5^ Department of Obstetrics and Gynaecology, School of Medicine, The University of Zambia, Lusaka, Zambia; ^6^ Health Sciences Library, University of North Carolina at Chapel Hill, Chapel Hill, NC, United States; ^7^ Department of Obstetrics and Gynecology, University of North Carolina at Chapel Hill, Chapel Hill, NC, United States

**Keywords:** hypertensive disorders, persistent hypertension, low- and middle-income countries, prevalence, pregnancy

## Abstract

**Background:**

Hypertensive disorders of pregnancy can lead to persistent hypertension (pHTN) in the months and even years following delivery. However, its prevalence in low- and middle-income countries (LMICs) is not well characterized.

**Objective:**

To synthesize available evidence on the pHTN prevalence following a pregnancy complicated by hypertensive disorders of pregnancy in LMICs.

**Search strategy:**

PubMed, CINAHL Plus, Global Health (EBSCO*host*), and Scopus from inception through a search date of July 12, 2022, and updated on January 2, 2024.

**Selection criteria:**

Cross-sectional studies and cohort studies reporting pHTN prevalence were eligible.

**Data collection and analysis:**

We conducted a narrative synthesis of data and categorized reported prevalence time points into several broader categories. We used the Newcastle-Ottawa checklist to assess the risk of bias. The protocol is registered in PROSPERO (CRD42022345739).

**Results:**

We reviewed 1,584 abstracts and identified 22 studies that reported pHTN between 2000 and 2023 from 14 LMICs. The overall prevalence of pHTN ranged between 6.9% and 62.2%, with the highest prevalence noted within African studies and the lowest in South American studies. Estimates at different follow-up periods postpartum were 6.9%–42.9% at six weeks, 34.0%–62.2% at three months, 14.8%–62.2% at six months, 12.7%–61.2% at 12 months, and 7.5%–31.8% at more than 12 months. The quality score of the selected studies ranged from 50% to 100%.

**Conclusions:**

The extant literature reports a high prevalence of pHTN in LMICs following a pregnancy complicated by hypertensive disorders. To reduce long-term complications of pHTN, programs should emphasize early screening and linkages to long-term care for at-risk women.

**Systematic Review Registration:**

https://www.crd.york.ac.uk/PROSPERO/display_record.php?RecordID=345739, PROSPERO (CRD42022345739)

## Background

Hypertension (HTN) is a metabolic condition characterized by elevated blood pressure (1). Nearly 1.3 billion adults globally have HTN, with less than half (42%) diagnosed and treated (2). A non-communicable disease, HTN is currently one of the main causes of death among men and women globally (3). Low- and middle-income countries (LMICs) bear a disproportionate burden due to unplanned rapid urbanization, population ageing and globalization of unhealthy lifestyles (2). Between 1990 and 2013, HTN-related deaths increased by 49%—to 10.4 million—a trend that is projected to continue (4). Hypertension can lead to cardiovascular diseases, including strokes and cardiac arrest, which are leading causes of premature deaths (5). Some risk factors for cardiovascular diseases are specific to women, and one of them is hypertensive disorders of pregnancy (HDP) (6).

Hypertensive disorders of pregnancy refer to gestational hypertension, preeclampsia or eclampsia (7–9). Preeclampsia and eclampsia are the most significant causes of maternal and perinatal morbidity and mortality after haemorrhage (10). Similarly, adverse events in neonates largely depend on the type and severity of the hypertensive disorder (11). Risk factors for developing HDP include but are not limited to a family history of hypertension, obesity, smoking, previous history of HDP, extremes of maternal age, alcohol use, smoking, and left ventricular hypertrophy (12).

Hypertensive disorders affect 5% to 10% of pregnant women globally and are increasing with the rise in cardiometabolic diseases in women of childbearing age (13). Studies have reported delayed recovery and persistent HDP symptoms that have been linked to the development of persistent hypertension (pHTN) long after giving birth (14, 15). HDP predisposes women to early cardiovascular diseases (3), which is why early diagnosis and follow-up of chronic hypertension are important in clinical management. While there have been several individual studies that reported pHTN, there are existing gaps. A recent systematic review and meta-analysis only assessed the severity of HDP and risk of cardiovascular diseases in different years after index pregnancy (16). A similar review focused on recurrent preeclampsia but did not assess gestational hypertension and eclampsia (17). However, we are unaware of systematic reviews on pHTN following a pregnancy complicated by gestational hypertension, preeclampsia and eclampsia in LMICs. Understanding the prevalence of pHTN by timing postpartum can inform in developing screening guidelines and promote linkage to HTN care among women of childbearing age. Through this systematic review, we sought to address this scientific gap.

## Methods

This review used the Preferred Reporting Items of Systematic Reviews and Meta-Analysis 2020 checklist (18). The protocol is registered in the International Prospective Register of Systematic Reviews PROSPERO (CRD42022345739).

## Eligibility criteria

This review included cross-sectional studies, retrospective and prospective cohort studies reporting pHTN burden after deliveries following a pregnancy complicated by HDP. Case-control studies, in which participants were selected based on the diagnosis of pHTN, were excluded to minimize bias. Only studies conducted in LMICs as defined by the World Bank (http://data.worldbank.org/about/country-and-lending-groups) were considered because this is where two-thirds of the global HTN cases occur (2), and monitoring of cardiovascular diseases is limited in these settings. We documented the criteria used within these selected studies to define gestational hypertension, preeclampsia and eclampsia. If the definition of HDP was not explicitly stated, we assumed that the definition used was derived from a widely used classification (7, 8, 19).

## Search strategy

We searched the following electronic bibliographic databases through the last search date of July 12, 2022, and updated on January 2, 2024: (PubMed, Scopus, Global Health (EBSCO*host*), and CINAHL Plus with Full Text (EBSCO*host*)) using a comprehensive search strategy (Supplementary Appendix S1). The search strategy was adapted for use across the different databases. No time restrictions were imposed; however, the searches were restricted to studies published in English as the research team members were only fluent in English. Furthermore, included articles and reference lists were screened for extra inclusion material.

## Data selection and abstraction

Search results from databases were downloaded to EndNote (Clarivate, Philadelphia, PA, USA), where duplicates were removed. Primary screening and data extraction was done using Covidence (Vertias Health Innovation, Melbourne, Australia), an internet-based systematic review study screening program. Two reviewers (MM and MKL) independently screened titles, abstracts, and full text for inclusion. Any discrepancies between the two reviewer’s summary reports were resolved through consensus. Data abstraction was conducted using a standardized data extraction form designed to capture variables of interest. Potentially relevant studies were retrieved in full, and a calibration exercise was conducted to assure clarity and non-duplication amongst reviewers. The following data were extracted from each study: country, study design, author, study period, sample sizes, publication year, follow-up period, source population, study setting/data source, the timing of HTN measurement, outcome measures (including definitions criteria), and effect measure estimates. Two reviewers (MM and MKL) conducted data abstraction, and any discrepancies were addressed through consensus. For any missing or unclear information, we contacted the corresponding authors at least three times if no response was received on the first attempt.

## Methodological approach

A PRISMA 2020 guideline for a systematic review and flow chart summarised the review’s search and selection of studies (20). We described the included articles and provided a summary of their characteristics. In addition, we reported estimates of the magnitude of pHTN with additional details about definitions and timing of measurements following a pregnancy complicated by HDP.

For pHTN, we assessed the proportion of women with a recent pregnancy complicated by HDP who remained hypertensive at the time of assessment. This review relied on the definitions of HTN used within individual studies. Estimates were reported with 95% confidence intervals (CIs). The estimates were reported at return visits and study endpoints for cohort studies. In addition, the total number of women with pHTN reported during a postpartum period was used for cross-sectional studies. We found that most studies did not clearly define all components of HDP, and assessment time points varied. Given this considerable heterogeneity, meta-analysis was deemed not feasible, and we proceeded with a narrative review alone (21).

## Methodological quality

Two independent reviewers (MM and MKL) used the Newcastle-Ottawa checklist to assess individual studies’ quality and risk of bias (22). Disagreement between two reviewers was resolved through consensus. Three domains were assessed: selection of participants, comparability of cohorts and outcome assessment. For the presence of a rating, a ‘yes’ was given one point; otherwise, a zero was assigned. The item scores were summed to obtain total scores. The percentage scores were calculated by dividing the article’s score by the maximum score possible and multiplying by 100. The total score ranged from 0 to 100%, with scores less than 65%, less than 76% and between 76% and 100% considered high, moderate and low-risk studies, respectively. We used the assessment of risk of bias to inform a sensitivity analysis that considered only studies with low overall risk of bias.

## Results

### Search results

**Figure 1:**
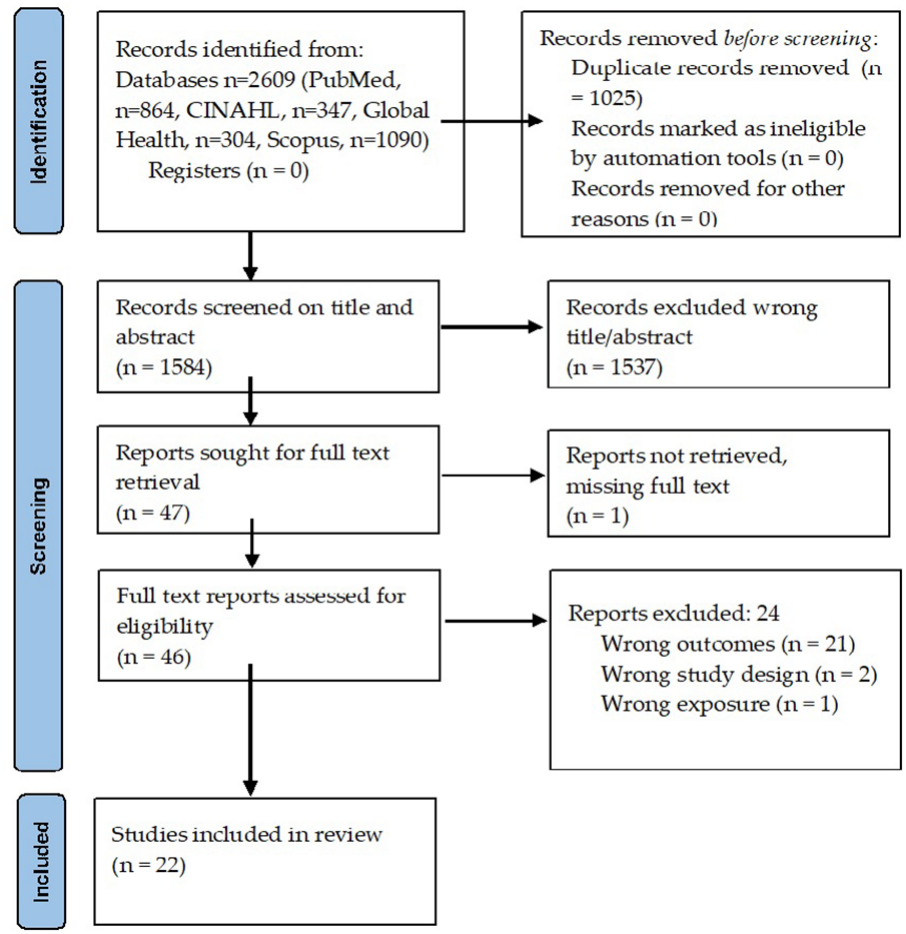
Summary of literature search and selection.

Overall, 2,609 articles were identified, with 1,025 duplicates removed (Figure 1). Full texts were obtained for 47 papers, of which 25 were excluded (Supplementary Table S1) upon further screening. The reasons for excluding studies were not being able to retrieve report (*n* = 1) (23), while the rest did not report pHTN as the primary outcome (*n* = 20) (24–43), wrong design (*n* = 2) (44, 45), oral presentation (*n* = 1) (46), or wrong exposure (*n* = 1) (47). After retrieval and full-text review, 22 published reports (Supplementary Table S1) were selected for this systematic review (14–16, 48–65).

## Characteristics of included studies

Characteristics of the studies are summarised in Table 1. Information regarding the prevalence of pHTN reported between 2000 and 2023 was obtained from 14 LMICs, with Uganda accounting for the largest proportion (18.2%), followed by Nigeria (13.6%) and Cameroon (13.6%). Fourteen studies were prospective cohorts (14, 15, 48–56, 64, 65), five were retrospective cohorts (57–61), and three were cross-sectional studies (16, 62, 63).

**Table 1:** Characteristics of included papers (*n* = 22).

Author and year	Country	Study design/study period	Timing of HTN measurement	Study setting/data source	Source population/HDP	HTN definition criteria	Sample size(*n*)	Effect measures
Amougou et al. (57)	Cameroon	Retrospective cohort 2009–2016	8 years	Records review	PE	Unclear	136	Persistent HTN 32/136 (23.5%)
Babah et al. (48)	Nigeria	Prospective cohort 2009	6 weeks	Labour wards	GH/PE	ACOG	29	Persistent HTN 3/29 (10.3%)
Cho et al. (58)	Korea	Retrospective cohort 2004–2012	8 years	Records review	PE	ISSHP	1,910	Persistent HTN 148/1,910 (7.7%)
Fadalallah et al. (15)	Sudan	Prospective cohort 2014	6 weeks	ANC	PE	ISSHP	165	Persistent HTN 58/165 (35.2%)
Tornes et al. (66)	Cuba	Prospective cohort 2017–2020	1 year	Labour wards	PE	ACOG	162	Persistent HTN 45/162 (27.8%)
Ishaku et al. (35)	Nigeria	Prospective cohort 2017–2018	9 weeks, 6 months and 1 year	ANC/Labour wards	GH/PE/EC	ISSHP	278	persistent HTN; GH = 64/278, 23% (9 weeks), 62/278, 22.3% (6 months), 62/278, 22.3% (12 months) PE/EC = 170/278, 62% (9 weeks), 170/278, 62% (6 months) and 173/278, 61.2% (12 months)
Kaze et al. (14)	Cameroon	Prospective cohort 2010–2012	6 weeks, 3 months and 6 months	ANC/Maternity unit	PE/EC	ISSHP	54	Persistent HTN 23/54, 42.6% (6 weeks), 15/54, 27.8% (3 months) 8/54, 14.8% (6 months)
Keepanasseril et al. (50)	India	Prospective cohort 2018–2019	3 months	Records review	PE	ACOG	32	Persistent HTN 32 (18.1%)
Lugobe et al. (64)	Uganda	Prospective cohort 2019	3 months	Labour ward	PE/EC/GH	Unclear	111	Persistent HTN 21/54 (39%)
Ma and Yao (51)	China	Prospective cohort 2014–2015	6 weeks	ANC	PE	ISSHP	173	Persistent HTN 61/173 (35.3%)
Mooij et al. (62)	Tanzania	Cross-sectional 2011–2018	7 years	Records review	PE	ISSHP	24	Persistent HTN 7/24 (29%)
Muteke et al. (65)	Uganda	2017–2018	6 weeks	Prospective cohort	PE	ACOG	85	Persistent HTN 5/73 (6.9%)
Nakimuli et al. (56)	Uganda	Prospective cohort 2009–2011	3 months	ANC/labour/postnatal ward	PE/EC	ISSHP	188	Persistent HTN 64/188 (34%)
Ndayambagye et al. (52)	Uganda	Prospective cohort 2008–2009	6 weeks	Labour/postnatal clinics	PE/EC	ISSHP	195	Persistent HTN 44/195 (27.7%)
Nganou-Gnindjio et al. (63)	Cameroon	Cross-sectional 2011–2016	6 months	Records review	PE/EC	Unclear	92	Persistent HTN 30/92 (32.6%)
Ntlemo et al. (55)	South Africa	Prospective cohort 2019–2020	6 weeks	ANC/labour wards	PE	ISSHP	150	Persistent HTN 49/150 (32.7%)
Olagbuji et al. (53)	Nigeria	Prospective cohort 2009–2010	6 weeks	Prenatal	GH/PE/EC	ISSHP	198	Persistent HTN 51/198 (25.8%)
Osoti et al. (54)	Kenya	Prospective cohort 2016–2018	6 months	ANC/labour wards	GH/PE	ISSHP	63	Persistent HTN 28/63 (44.4%)
Shahbazian et al. (59)	Iran	Retrospective cohort 2001–2003	5 years	Records review	PE	ISSHP	35	Persistent HTN 10/35 (28.6%)
Shammas (60)	Jordan	Retrospective cohort 1988–1998	10 years	Records review	PE/GH	Unclear	101	Persistent HTN 11/47 (23%) for PE, 21/54 (39%) for GH
Sukmanee et al. (16)	Thailand	Nested cross-sectional 2014–2020	7 years	Records review	PE	ISSHP	88	Persistent HTN 28/88 (31.8%)
Wang et al. (61)	China	Retrospective cohort 2014–2020	2 years	Records review	PE/GH/EC	ESH-ESC	1,261	Persistent HTN 94/1,261 (7.5%)

PE, preeclampsia; GH, Gestational hypertension; EC, Eclapmsia; HTN, hypertension; HDP, hypertensive disorders of pregnancy; ANC, antenatal care; ISSHP, International Society for the Study of Hypertension in Pregnancy; ACOG, American College of Obstetrician and Gynaecologists; ESH–ESC, European Society of Hypertension–European Society of Cardiology.

For the definitions of HTN, 13 studies used the International Society for the Study of Hypertension in Pregnancy recommendations (8), four used the American College of Obstetricians and Gynecologists (7), one used the European Society of Hypertension–European Society of Cardiology, and four did not mention criteria used (Table 1).

We noted considerable variability from selected studies concerning HTN definition, study design, study setting/data source and follow-up period post-delivery. Preeclampsia as a component of HDP was reported in all 22 studies. However, only seven studies reported gestational hypertension, and eight studies reported eclampsia (Table 1).

## Persistent hypertension

**Figure 2:**
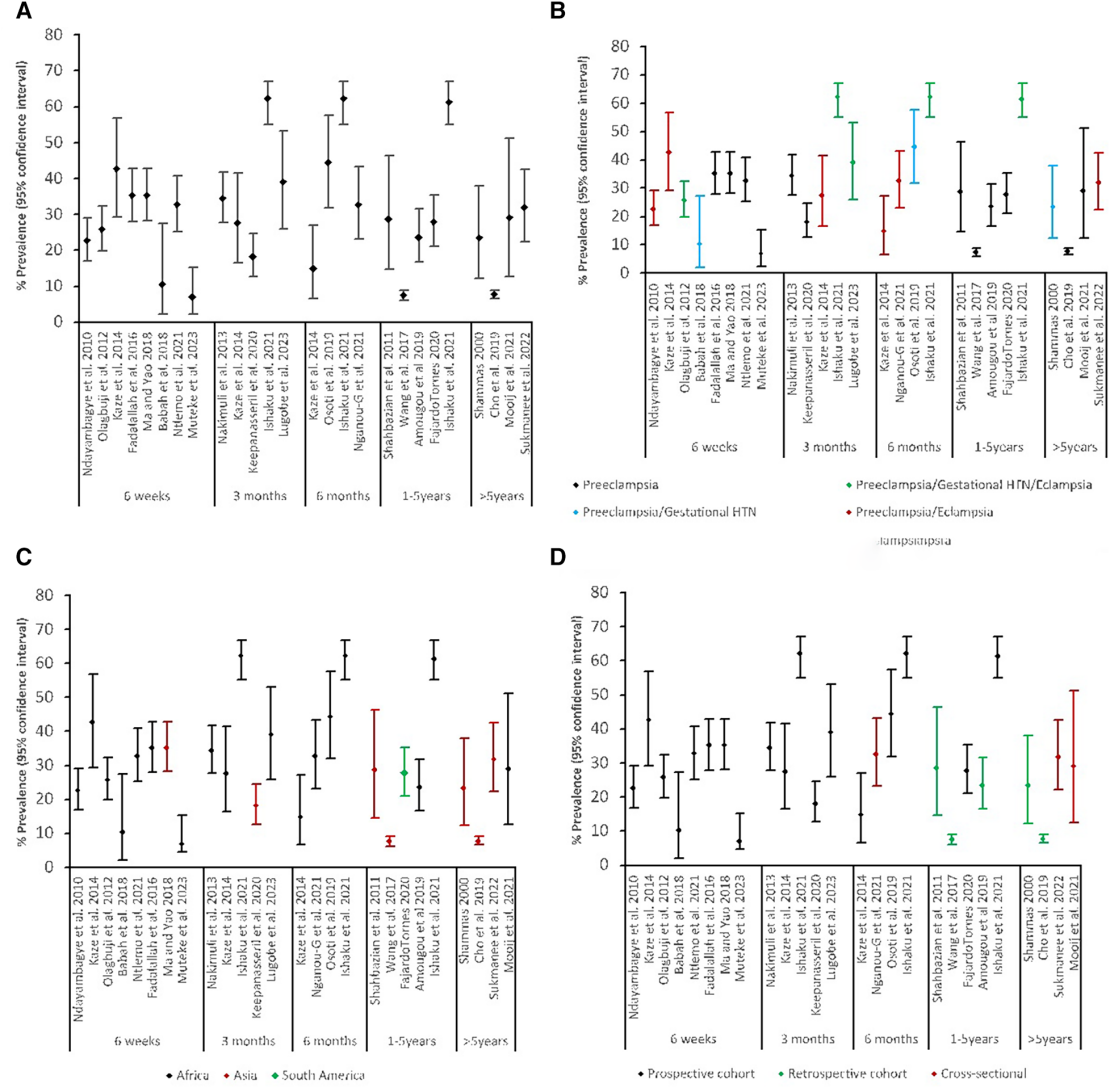
Prevalence of persistent HTN: (**A**) by timing of measurement, (**B**) by timing of measurement and source population, (**C**) by timing of measurement and region, (**D**) by timing of measurement and study design.

All 22 studies reported pHTN post-delivery of pregnancy complicated by HDP, ranging between 6.9% and 62.2% (Table 1). There was high heterogeneity with the timing of HTN measurement reported as an interval from delivery until the development of HTN at the time of assessment. We categorized these time points into several broader categories: six weeks, three months, six months, 12 months and more than 12 months to examine the prevalence of pHTN. The ranges reported were 6.9%–42.9% at six weeks, 34.0%–62.2% at three months, 14.8%–62.2% at six months, 12.7%–61.2% at 12 months, and 7.5%–31.8% at more than 12 months. The prevalences are shown in Figure 2A.

The prevalence varied considerably by region of LMICs (Figure 2C). The prevalence of pHTN in the African region was highest at any given time, followed by South America, and Asia was the least. Figure 2B shows the types of HDP considered within the study populations. Studies that reported all three hypertensive disorders had higher rates of pHTN at any time, followed by combined preeclampsia and eclampsia. When stratified by the study design (Figure 2D), prospective cohort studies reported higher prevalence rates (10.3%–62.2%) than retrospective (7.2%–31.6%) and cross-sectional studies (23.2%–42.2%), regardless of the source population.

## Quality appraisal

Twenty studies were included in quality appraisals (Table 2). Two studies were excluded from the quality appraisal because they reported pHTN as part of the composite outcome. The risk of bias was low in 17 studies, moderate in one study and high in four studies. Most studies were considered to be at high risk of bias because they did not have a non-exposed group. Overall, the quality score of the selected studies ranged from 50% to 100%.

**Table 2:** Risk of bias assessment of included studies with persistent hypertension as a primary outcome (*n* = 20).

Author and year	Selection				Comparability	Outcome			Overall score	Risk of bias
	Exposed representation	Selection of non-exposed group	Ascertainment of HDP	At the study start outcome was absent	Adjustment for age and BMI	Outcome assessment	Follow up length	Adequacy of follow up		
Amougou et al. (57)	Yes	–	–	Yes	Yes	–	Yes	Yes	5/8 (62.5%)	High
Babah et al. (48)	Yes	Yes	Yes	Yes	Yes	Yes	Yes	Yes	8/8 (100%)	Low
Cho et al. (58)	Yes	–	Yes	Yes	Yes	–	Yes	Yes	6/8 (75.0%)	Moderate
Fadalallah et al. (15)	Yes	–	Yes	Yes	Yes	Yes	Yes	Yes	7/8 (87.5%)	Low
Tornes et al. (66)	Yes	–	Yes	Yes	Yes	Yes	Yes	Yes	7/8 (87.5%)	Low
Ishaku et al. (35)	Yes	–	Yes	Yes	Yes	Yes	Yes	Yes	7/8 (87.5%)	Low
Kaze et al. (14)	Yes		Yes	Yes	Yes	Yes	Yes	Yes	7/8 (87.5%)	Low
Keepanasseril et al. (50)	Yes	–	Yes	Yes	Yes	Yes	Yes	Yes	7/8 (87.5%)	Low
Lugobe et al. (64)	Yes	–	Yes	Yes	Yes	Yes	Yes	Yes	7/8 (87.5%)	Low
Ma and Yao (51)	Yes	–	Yes	Yes	Yes	Yes	Yes	Yes	7/8 (87.5%)	Low
Mooij et al. (62)	Yes	Yes	Yes	–	Yes	Yes	–	–	5/8 (62.5%)	High
Muteke et al. (65)	Yes	–	Yes	Yes	Yes	Yes	Yes	Yes	7/8 (87.5%)	Low
Nakimuli et al. (56)	Yes	–	Yes	Yes	Yes	Yes	Yes	Yes	7/8 (87.5%)	Low
Ndayambagye et al. (52)	Yes	–	Yes	Yes	Yes	Yes	Yes	Yes	7/8 (87.5%)	Low
Nganou-Gnindjio et al. (63)	Yes	–	Yes	–	Yes	Yes	–	–	4/8 (50.0%)	High
Olagbuji et al. (53)	Yes	–	Yes	Yes	Yes	Yes	Yes	Yes	7/8 (87.5%)	Low
Shahbazian et al. (59)	Yes	Yes	Yes	Yes	Yes	Yes	Yes	Yes	7/8 (87.5%)	Low
Shammas (60)	Yes	Yes	Yes	Yes	–	Yes	Yes	Yes	7/8 (87.5%)	Low
Sukmanee et al. (16)	Yes	Yes	Yes	–	Yes	Yes	–	–	5/8 (62.5%)	High
Wang et al. (61)	Yes	Yes	Yes	Yes	Yes	Yes	Yes	Yes	8/8 (100%)	Low

## Sensitivity analysis

In sensitivity analysis, including only studies with a low risk of methodological bias, the overall prevalence of pHTN ranged from 6.9%–62.2%. The prevalence was less varied 7.5%–23.5% at more than 12 months.

## Discussion

This systematic review aimed to examine the prevalence of pHTN among women with prior HDP in LMICs. We found that the prevalence of pHTN post-delivery varied greatly among women diagnosed with HDP in this region. The prevalence appeared to differ by region, type of HDP, timing of HTN measurement postpartum, and study design—but remained high throughout.

Among the included studies, there was a high variation in the prevalence of pHTN. Large proportions of women with HDP had high blood pressure six weeks after delivery, with the longest follow-up period being ten years. This was consistent with prior systematic reviews that included only preeclampsia as a source population (17, 67). In this systematic review, studies that included all HDP in the source population reported the highest prevalence rates of pHTN after pregnancy, followed by those with combined preeclampsia and eclampsia, and the least was preeclampsia. The mechanism explaining the relation between pHTN and HDP is considered multifactorial and challenging to study if thresholds are different and there is no standard approach. Studies have shown gestational hypertension and preeclampsia to be independent risk factors after adjusting for known cardiovascular risk markers like obesity and advanced age (68). A meta-analysis by Heida and colleagues (69) found that the risk of pHTN after delivery among women with gestational hypertension was comparable to women with preeclampsia. Other mechanisms that play a role in gestational hypertension or preeclampsia are of an inflammatory nature (70). Gestational hypertension and preeclampsia have been linked to elevated inflammatory biomarkers during pregnancy (71, 72), providing biological plausibility for persistent changes after pregnancy. Therefore, inflammatory biomarkers may provide insight into the development of pHTN after complicated pregnancies. Furthermore, at the time of diagnosis, pregnant women with gestational hypertension and preeclampsia have profound systemic inflammation and disruption of the endothelium (73). This suggests that pregnancy could be an early stress test providing an opportunity to identify women at risk of HTN early in life (37).

Among the studies in this review, the prevalence of pHTN was high. The prevalence was highest in African countries like Nigeria (49) and Kenya (54) and the lowest in South America (Cuba) (66) and Asia (China) (51), regardless of the timing of measurement. In high-income countries, the rates are lower. For instance, in the United Kingdom, they reported 13% (74), in the USA, 58.6% (75) and 3% in Australia (76). It is plausible that the variability in sociodemographic factors, maternal risk factors, and differences in postpartum care service utilization across the LMICs might explain the observed results. For instance, in sub-Saharan Africa, women often present late and with advanced disease states due to poor health-seeking behaviour, contributing to high co-morbidity and mortality (77, 78). Other plausible explanations for the observed estimates may be attributable to genetic differences between African populations and other regions (79), but the quality of care social and structural determinants of health have also been proposed as contributing factors (80).

Persistent hypertension prevalence rates depended on the timing of its measurement. Although HDP typically resolves in the window following delivery, its effects remain in the postpartum period due to the delay for most body systems to return to norma**l** (81). It is possible that women at different time points do not have persistent long-term hypertension but are still resolving their HDP. However, this is less likely to be a problem over the course of follow-up, especially at the 12- and 24-month time points. The timing of hypertension screening is key to developing clinical guidelines for postpartum care for women with prior HDP. These findings suggest that, in LMICs, services to screen and treat HTN may be needed for women with HDP. In middle and high-income countries, several guidelines have been developed regarding pHTN post-delivery abeit inconsistent on the intervention thresholds. Some guidelines recommend initiating follow-up at 6–8 weeks post-delivery (82, 83), whereas others advise starting 6–12 months post-delivery (7, 84, 85). There is no consensus approach in many national programs, and the result is a non-standardized approach that may be difficult to implement. The observed regional and between-country disparities highlight the need, following delivery, to monitor women with HDP to ensure their well-being and prevent complications (8).

## Strengths and limitations

The strength of this analysis was that, to the best of our knowledge, regional estimates of pHTN in LMICs have not been previously reported. The results from this systematic review challenge the status quo in most LMICs where women are mostly discharged and never followed up beyond six weeks after giving birth. Despite the strengths of this systematic review, these findings should be interpreted in the context of some limitations. The present systematic review excluded all sources written in languages other than English. In addition, there are several limitations to this combination of study designs, including the use of a checklist for quality appraisal and different guidelines, which can account for a large part of the variations in reported pHTN rates in the reviewed studies. Furthermore, the small number of studies from some regions of LMICs, such as South America, may have affected the true estimates for this region.

## Conclusion

The present systematic review provided a narrative synthesis of available evidence regarding pHTN in LMICs following a pregnancy complicated by HDP. The prevalence appeared heterogeneous across the region due in part to the varied study timing and source populations.

## Implications

Rates of pHTN appeared high across multiple settings and populations, suggesting that standard approaches to HTN screening, care, and treatment may be needed for women with HDP. Future research should focus on the design of such services, weighing both their costs and long-term benefits.

## Data Availability

The original contributions presented in the study are included in the article/Supplementary Material, further inquiries can be directed to the corresponding author.
