# Supplementary material for: Prevalence of persistent hypertension following pregnancy complicated by hypertensive disorders in low- and middle-income countries: a systematic review
Source: Front Glob Womens Health. 2024 Mar 1;5:1315763. doi: 10.3389/fgwh.2024.1315763 (PMC10940323; doi:10.3389/fgwh.2024.1315763)
Supplement: Supplementary file 2 [file Datasheet1.pdf]

Search Strategy Report:

Date: 02/01/2024

Total # of References: 2609
# of Duplicates Removed: 1025
Total # of References to Screen: 1584

Database: PubMed

Set #		Results
1	"Hypertension"[Mesh] OR hypertension[tiab] OR hypertensive[tiab] OR “high blood pressure”[tiab] OR “high blood pressures”[tiab]	
2	"Hypertension, Pregnancy-Induced"[Mesh] OR eclampsia[tiab] OR eclampsias[tiab] OR preeclampsia[tiab] OR preeclampsias[tiab] OR “HELLP Syndrome”[tiab] OR “pregnancy induced”[tiab] OR “pregnancy transient hypertension”[tiab] OR “gestational hypertension”[tiab]	
3	"Postpartum Period"[Mesh] OR postpartum[tiab] OR postnatal[tiab] OR post-pregnancy[tiab] OR Puerperium[tiab] OR persistent[tiab] OR persisting[tiab] OR persisted[tiab] OR persists[tiab] OR persist[tiab] OR persistence[tiab] OR chronic[tiab]	
4	(“Deprived Countries”[tw] OR “Deprived Population”[tw] OR “Deprived Populations”[tw] OR “Developing Countries”[tw] OR “Developing Country“[tw] OR “Developing Economies“[tw] OR “Developing Economy”[tw] OR “Developing Nation”[tw] OR “Developing Nations”[tw] OR “Developing Population”[tw] OR “Developing Populations”[tw] OR “Developing World”[tw] OR “LAMI Countries”[tw] OR “LAMI Country”[tw] OR “Less Developed Countries”[tw] OR “Less Developed Country”[tw] OR “Less Developed Economies“[tw] OR “Less Developed Nation”[tw] OR “Less Developed Nations”[tw] OR “Less Developed World”[tw] OR “Lesser Developed Countries”[tw] OR “Lesser Developed Nations”[tw] OR LMIC[tw] OR LMICS[tw] OR “Low GDP”[tw] OR “Low GNP”[tw] OR “Low Gross Domestic”[tw] OR “Low Gross National”[tw] OR “Low Income Countries”[tw] OR “Low Income Country”[tw] OR “Low Income Economies“[tw] OR “Low Income Economy”[tw] OR “Low Income Nations”[tw] OR “Low Income Population”[tw] OR “Low Income Populations”[tw] OR “Lower GDP”[tw] OR “lower gross domestic”[tw] OR “Lower Income Countries”[tw] OR “Lower Income Country”[tw] OR “Lower Income Nations”[tw] OR “Lower Income Population”[tw] OR “Lower Income Populations”[tw] OR “Middle Income Countries”[tw] OR “Middle Income Country”[tw] OR “Middle Income Economies“[tw] OR “Middle Income Nation”[tw] OR “Middle Income Nations”[tw] OR “Middle Income Population”[tw] OR “Middle Income Populations”[tw] OR “Poor Countries”[tw] OR “Poor Country”[tw] OR “ Poor Economies“[tw] OR “Poor Economy”[tw] OR “Poor Nation”[tw] OR “Poor Nations”[tw] OR “Poor Population”[tw] OR “Poor Populations”[tw] OR “poor world”[tw] OR “Poorer Countries”[tw] OR “Poorer Economies“[tw] OR “Poorer Economy”[tw] OR “Poorer Nations”[tw] OR “Poorer Population”[tw] OR “ Poorer Populations”[tw] OR “Third World”[tw] OR “Transitional Countries”[tw] OR “Transitional Country”[tw] OR “Transitional Economies”[tw] OR “Transitional Economy”[tw] OR “Under Developed Countries”[tw] OR	

	“Under Developed Country”[tw] OR “under developed nations”[tw] OR “Under Developed World”[tw] OR “Under Served Population”[tw] OR “Under Served Populations”[tw] OR “Underdeveloped Countries”[tw] OR “Underdeveloped Country”[tw] OR “underdeveloped economies”[tw] OR “underdeveloped nations”[tw] OR “underdeveloped population”[tw] OR “Underdeveloped World”[tw] OR “Underserved Countries”[tw] OR “Underserved Nations”[tw] OR “Underserved Population”[tw] OR “Underserved Populations”[tw] OR Afghanistan[tw] OR Albania[tw] OR Algeria[tw] OR “American Samoa”[tw] OR Angola[tw] OR Argentina[tw] OR “Argentine Republic”[tw] OR Armenia[tw] OR Azerbaijan[tw] OR Bangladesh[tw] OR Belarus[tw] OR Byelarus[tw] OR Belorussia[tw] OR Belize[tw] OR Benin[tw] OR Bhutan[tw] OR Bolivia[tw] OR Bosnia[tw] OR Botswana[tw] OR Brazil[tw] OR Bulgaria[tw] OR Burma[tw] OR “Burkina Faso”[tw] OR Burundi[tw] OR “Cabo Verde”[tw] OR “Cape verde”[tw] OR Cambodia[tw] OR Cameroon[tw] OR “Central African Republic”[tw] OR Chad[tw] OR China[tw] OR Colombia[tw] OR Comoros[tw] OR Comores[tw] OR Comoro[tw] OR Congo[tw] OR “Costa Rica”[tw] OR “Côte d'Ivoire”[tw] OR Cuba[tw] OR Djibouti[tw] OR Dominica[tw] OR “Dominican Republic”[tw] OR Ecuador[tw] OR Egypt[tw] OR “El Salvador”[tw] OR Eritrea[tw] OR Ethiopia[tw] OR Fiji[tw] OR Gabon[tw] OR Gambia[tw] OR Gaza[tw] OR “Georgia Republic”[tw] OR Georgian[tw] OR Ghana[tw] OR Grenada[tw] OR Grenadines[tw] OR Guatemala[tw] OR Guinea[tw] OR “Guinea Bissau”[tw] OR Guyana[tw] OR Haiti[tw] OR Herzegovina[tw] OR Hercegovina[tw] OR Honduras[tw] OR India[tw] OR Indonesia[tw] OR Iran[tw] OR Iraq[tw] OR Jamaica[tw] OR Jordan[tw] OR Kazakhstan[tw] OR Kenya[tw] OR Kiribati[tw] OR Korea[tw] OR Kosovo[tw] OR Kyrgyz[tw] OR Kirghizia[tw] OR Kirghiz[tw] OR Kirgizstan[tw] OR Kyrgyzstan[tw] OR “Lao PDR”[tw] OR Laos[tw] OR Lebanon[tw] OR Lesotho[tw] OR Liberia[tw] OR Libya[tw] OR Macedonia[tw] OR Madagascar[tw] OR Malawi[tw] OR Malay[tw] OR Malaya[tw] OR Malaysia[tw] OR Maldives[tw] OR Mali[tw] OR “Marshall Islands”[tw] OR Mauritania[tw] OR Mauritius[tw] OR Mexico[tw] OR Micronesia[tw] OR Moldova[tw] OR Mongolia[tw] OR Montenegro[tw] OR Morocco[tw] OR Mozambique[tw] OR Myanmar[tw] OR Namibia[tw] OR Nauru[tw] OR Nepal[tw] OR Nicaragua[tw] OR Niger[tw] OR Nigeria [tw] OR Pakistan [tw] OR Palau[tw] OR Panama[tw] OR “Papua New Guinea”[tw] OR Paraguay[tw] OR Peru [tw] OR Philippines[tw] OR Phillippines[tw] OR Philipines[tw] OR Phillipines[tw] OR Principe[tw] OR Romania[tw] OR Rwanda[tw] OR Ruanda[tw] OR Samoa[tw] OR “Sao Tome”[tw] OR Senegal[tw] OR Serbia[tw] OR “Sierra Leone”[tw] OR “Solomon Islands”[tw] OR Somalia[tw] OR “South Africa”[tw] OR “South Sudan”[tw] OR “Sri Lanka”[tw] OR “St Lucia”[tw] OR “St Vincent”[tw] OR Sudan[tw] OR Surinam[tw] OR Suriname[tw] OR Swaziland[tw] OR Syria[tw] OR “Syrian Arab Republic”[tw] OR Tajikistan[tw] OR Tadzhikistan[tw] OR Tadjikistan[tw] OR Tadzhik[tw] OR Tanzania[tw] OR Thailand[tw] OR Timor[tw] OR Togo[tw] OR Tonga[tw] OR Tunisia[tw] OR Turkey[tw] OR Turkmen[tw] OR Turkmenistan[tw] OR Tuvalu[tw] OR Uganda[tw] OR Ukraine[tw] OR Uzbek[tw] OR Uzbekistan[tw] OR Vanuatu[tw] OR Venezuela[tw] OR Vietnam[tw] OR “West Bank”[tw] OR Yemen[tw] OR Zambia[tw] OR Zimbabwe[tw])	
5	#1 AND #2 AND #3 AND #4	
6	#5 AND English[lang]	587

Database: CINAHL Plus with Full Text (EBSCO*host*)

Set #		Results
1	MH "Hypertension+" OR TI (hypertension OR hypertensive OR “high blood pressure” OR “high blood pressures”) OR AB (hypertension OR hypertensive OR “high blood pressure” OR “high blood pressures”)	
2	MH "Pregnancy-Induced Hypertension+" OR TI (eclampsia OR eclampsias OR preeclampsia OR preeclampsias OR “HELLP Syndrome” OR “pregnancy induced” OR “pregnancy transient hypertension” OR “gestational hypertension”) OR AB (eclampsia OR eclampsias OR preeclampsia OR preeclampsias OR “HELLP Syndrome” OR “pregnancy induced” OR “pregnancy transient hypertension” OR “gestational hypertension”)	
3	MH "Postnatal Period+" OR TI (postpartum OR postnatal OR post-pregnancy OR Puerperium OR persistent OR persisting OR persisted OR persists OR persist OR persistence OR chronic) OR AB (postpartum OR postnatal OR post-pregnancy OR Puerperium OR persistent OR persisting OR persisted OR persists OR persist OR persistence OR chronic)	
4	(“Deprived Countries” OR “Deprived Population” OR “Deprived Populations” OR “Developing Countries” OR “Developing Country“ OR “Developing Economies“ OR “Developing Economy” OR “Developing Nation” OR “Developing Nations” OR “Developing Population” OR “Developing Populations” OR “Developing World” OR “LAMI Countries” OR “LAMI Country” OR “Less Developed Countries” OR “Less Developed Country” OR “Less Developed Economies“ OR “Less Developed Nation” OR “Less Developed Nations” OR “Less Developed World” OR “Lesser Developed Countries” OR “Lesser Developed Nations” OR LMIC OR LMICS OR “Low GDP” OR “Low GNP” OR “Low Gross Domestic” OR “Low Gross National” OR “Low Income Countries” OR “Low Income Country” OR “Low Income Economies“ OR “Low Income Economy” OR “Low Income Nations” OR “Low Income Population” OR “Low Income Populations” OR “Lower GDP” OR “lower gross domestic” OR “Lower Income Countries” OR “Lower Income Country” OR “Lower Income Nations” OR “Lower Income Population” OR “Lower Income Populations” OR “Middle Income Countries” OR “Middle Income Country” OR “Middle Income Economies“ OR “Middle Income Nation” OR “Middle Income Nations” OR “Middle Income Population” OR “Middle Income Populations” OR “Poor Countries” OR “Poor Country” OR “ Poor Economies“ OR “Poor Economy” OR “Poor Nation” OR “Poor Nations” OR “Poor Population” OR “Poor Populations” OR “poor world” OR “Poorer Countries” OR “Poorer Economies“ OR “Poorer Economy” OR “Poorer Nations” OR “Poorer Population” OR “ Poorer Populations” OR “Third World” OR “Transitional Countries” OR “Transitional Country” OR “Transitional Economies” OR “Transitional Economy” OR “Under Developed Countries” OR “Under Developed Country” OR “under developed nations” OR “Under Developed World” OR “Under Served Population” OR “Under Served Populations” OR “Underdeveloped Countries” OR “Underdeveloped Country” OR “underdeveloped economies” OR “underdeveloped nations” OR “underdeveloped	

	population" OR "Underdeveloped World" OR "Underserved Countries" OR "Underserved Nations" OR "Underserved Population" OR "Underserved Populations" OR Afghanistan OR Albania OR Algeria OR "American Samoa" OR Angola OR Argentina OR "Argentine Republic" OR Armenia OR Azerbaijan OR Bangladesh OR Belarus OR Byelarus OR Belorussia OR Belize OR Benin OR Bhutan OR Bolivia OR Bosnia OR Botswana OR Brazil OR Bulgaria OR Burma OR "Burkina Faso" OR Burundi OR "Cabo Verde" OR "Cape verde" OR Cambodia OR Cameroon OR "Central African Republic" OR Chad OR China OR Colombia OR Comoros OR Comores OR Comoro OR Congo OR "Costa Rica" OR "Côte d'Ivoire" OR Cuba OR Djibouti OR Dominica OR "Dominican Republic" OR Ecuador OR Egypt OR "El Salvador" OR Eritrea OR Ethiopia OR Fiji OR Gabon OR Gambia OR Gaza OR "Georgia Republic" OR Georgian OR Ghana OR Grenada OR Grenadines OR Guatemala OR Guinea OR "Guinea Bissau" OR Guyana OR Haiti OR Herzegovina OR Hercegovina OR Honduras OR India OR Indonesia OR Iran OR Iraq OR Jamaica OR Jordan OR Kazakhstan OR Kenya OR Kiribati OR Korea OR Kosovo OR Kyrgyz OR Kirghizia OR Kirghiz OR Kirgizstan OR Kyrgyzstan OR "Lao PDR" OR Laos OR Lebanon OR Lesotho OR Liberia OR Libya OR Macedonia OR Madagascar OR Malawi OR Malay OR Malaya OR Malaysia OR Maldives OR Mali OR "Marshall Islands" OR Mauritania OR Mauritius OR Mexico OR Micronesia OR Moldova OR Mongolia OR Montenegro OR Morocco OR Mozambique OR Myanmar OR Namibia OR Nauru OR Nepal OR Nicaragua OR Niger OR Nigeria OR Pakistan OR Palau OR Panama OR "Papua New Guinea" OR Paraguay OR Peru OR Philippines OR Phillippines OR Philipines OR Phillipines OR Principe OR Romania OR Rwanda OR Ruanda OR Samoa OR "Sao Tome" OR Senegal OR Serbia OR "Sierra Leone" OR "Solomon Islands" OR Somalia OR "South Africa" OR "South Sudan" OR "Sri Lanka" OR "St Lucia" OR "St Vincent" OR Sudan OR Surinam OR Suriname OR Swaziland OR Syria OR "Syrian Arab Republic" OR Tajikistan OR Tadzhikistan OR Tadjikistan OR Tadzhik OR Tanzania OR Thailand OR Timor OR Togo OR Tonga OR Tunisia OR Turkey OR Turkmen OR Turkmenistan OR Tuvalu OR Uganda OR Ukraine OR Uzbek OR Uzbekistan OR Vanuatu OR Venezuela OR Vietnam OR "West Bank" OR Yemen OR Zambia OR Zimbabwe)	
5	#1 AND #2 AND #3 AND #4	
6	#5 AND Narrow by Language: - english	302

Database: Global Health (EBSCO*host*)

Set #		Results
1	DE "hypertension" OR DE "portal hypertension" OR TI (hypertension OR hypertensive OR "high blood pressure" OR "high blood pressures") OR AB (hypertension OR hypertensive OR "high blood pressure" OR "high blood pressures")	
2	TI (eclampsia OR eclampsias OR preeclampsia OR preeclampsias OR "HELLP Syndrome" OR "pregnancy induced" OR "pregnancy transient hypertension" OR "gestational hypertension") OR AB (eclampsia OR eclampsias OR preeclampsia OR preeclampsias OR "HELLP Syndrome" OR "pregnancy induced" OR "pregnancy transient hypertension" OR "gestational hypertension")	

3	DE "puerperium" OR TI (postpartum OR postnatal OR post-pregnancy OR Puerperium OR persistent OR persisting OR persisted OR persists OR persist OR persistence OR chronic) OR AB (postpartum OR postnatal OR post-pregnancy OR Puerperium OR persistent OR persisting OR persisted OR persists OR persist OR persistence OR chronic)	
4	(“Deprived Countries” OR “Deprived Population” OR “Deprived Populations” OR “Developing Countries” OR “Developing Country“ OR “Developing Economies“ OR “Developing Economy” OR “Developing Nation” OR “Developing Nations” OR “Developing Population” OR “Developing Populations” OR “Developing World” OR “LAMI Countries” OR “LAMI Country” OR “Less Developed Countries” OR “Less Developed Country” OR “Less Developed Economies“ OR “Less Developed Nation” OR “Less Developed Nations” OR “Less Developed World” OR “Lesser Developed Countries” OR “Lesser Developed Nations” OR LMIC OR LMICS OR “Low GDP” OR “Low GNP” OR “Low Gross Domestic” OR “Low Gross National” OR “Low Income Countries” OR “Low Income Country” OR “Low Income Economies“ OR “Low Income Economy” OR “Low Income Nations” OR “Low Income Population” OR “Low Income Populations” OR “Lower GDP” OR “lower gross domestic” OR “Lower Income Countries” OR “Lower Income Country” OR “Lower Income Nations” OR “Lower Income Population” OR “Lower Income Populations” OR “Middle Income Countries” OR “Middle Income Country” OR “Middle Income Economies“ OR “Middle Income Nation” OR “Middle Income Nations” OR “Middle Income Population” OR “Middle Income Populations” OR “Poor Countries” OR “Poor Country” OR “ Poor Economies“ OR “Poor Economy” OR “Poor Nation” OR “Poor Nations” OR “Poor Population” OR “Poor Populations” OR “poor world” OR “Poorer Countries” OR “Poorer Economies“ OR “Poorer Economy” OR “Poorer Nations” OR “Poorer Population” OR “ Poorer Populations” OR “Third World” OR “Transitional Countries” OR “Transitional Country” OR “Transitional Economies” OR “Transitional Economy” OR “Under Developed Countries” OR “Under Developed Country” OR “under developed nations” OR “Under Developed World” OR “Under Served Population” OR “Under Served Populations” OR “Underdeveloped Countries” OR “Underdeveloped Country” OR “underdeveloped economies” OR “underdeveloped nations” OR “underdeveloped population” OR “Underdeveloped World” OR “Underserved Countries” OR “Underserved Nations” OR “Underserved Population” OR “Underserved Populations” OR Afghanistan OR Albania OR Algeria OR “American Samoa” OR Angola OR Argentina OR “Argentine Republic” OR Armenia OR Azerbaijan OR Bangladesh OR Belarus OR Byelarus OR Belorussia OR Belize OR Benin OR Bhutan OR Bolivia OR Bosnia OR Botswana OR Brazil OR Bulgaria OR Burma OR “Burkina Faso” OR Burundi OR “Cabo Verde” OR “Cape verde” OR Cambodia OR Cameroon OR “Central African Republic” OR Chad OR China OR Colombia OR Comoros OR Comores OR Comoro OR Congo OR “Costa Rica” OR “Côte d'Ivoire” OR Cuba OR Djibouti OR Dominica OR “Dominican Republic” OR Ecuador OR Egypt OR “El Salvador” OR Eritrea OR Ethiopia OR Fiji OR Gabon OR Gambia OR Gaza OR “Georgia Republic” OR Georgian OR Ghana OR Grenada OR Grenadines OR Guatemala OR Guinea OR “Guinea Bissau” OR Guyana OR Haiti OR Herzegovina OR Hercegovina OR Honduras OR India OR Indonesia OR Iran OR Iraq OR Jamaica OR Jordan OR Kazakhstan OR Kenya OR Kiribati OR Korea OR Kosovo OR Kyrgyz OR Kirghizia OR Kirghiz OR Kirgizstan OR Kyrgyzstan OR “Lao PDR” OR Laos OR Lebanon OR Lesotho OR Liberia OR Libya OR Macedonia OR Madagascar OR Malawi OR Malay OR Malaya OR Malaysia OR Maldives OR Mali OR “Marshall Islands” OR Mauritania OR Mauritius OR Mexico OR Micronesia OR Moldova OR Mongolia OR Montenegro OR Morocco OR Mozambique OR Myanmar OR Namibia OR	

	Nauru OR Nepal OR Nicaragua OR Niger OR Nigeria OR Pakistan OR Palau OR Panama OR “Papua New Guinea” OR Paraguay OR Peru OR Philippines OR Phillippines OR Philipines OR Phillipines OR Principe OR Romania OR Rwanda OR Ruanda OR Samoa OR “Sao Tome” OR Senegal OR Serbia OR “Sierra Leone” OR “Solomon Islands” OR Somalia OR “South Africa” OR “South Sudan” OR “Sri Lanka” OR “St Lucia” OR “St Vincent” OR Sudan OR Surinam OR Suriname OR Swaziland OR Syria OR “Syrian Arab Republic” OR Tajikistan OR Tadzhikistan OR Tadjikistan OR Tadzhik OR Tanzania OR Thailand OR Timor OR Togo OR Tonga OR Tunisia OR Turkey OR Turkmen OR Turkmenistan OR Tuvalu OR Uganda OR Ukraine OR Uzbek OR Uzbekistan OR Vanuatu OR Venezuela OR Vietnam OR “West Bank” OR Yemen OR Zambia OR Zimbabwe)	
5	#1 AND #2 AND #3 AND #4	
6	#5 AND Narrow by Language: - english	269

Database: Scopus (Elsevier)

Set #		Results
1	TITLE-ABS-KEY (hypertension OR hypertensive OR "high blood pressure" OR "high blood pressures")	
2	TITLE-ABS-KEY (eclampsia OR eclampsias OR preeclampsia OR preeclampsias OR "HELLP Syndrome" OR "pregnancy induced" OR "pregnancy transient hypertension" OR "gestational hypertension")	
3	TITLE-ABS-KEY (postpartum OR postnatal OR post-pregnancy OR puerperium OR persistent OR persisting OR persisted OR persists OR persist OR persistence OR chronic)	
4	TITLE-ABS-KEY ("Deprived Countries" OR "Deprived Population" OR "Deprived Populations" OR "Developing Countries" OR "Developing Country" OR "Developing Economies" OR "Developing Economy" OR "Developing Nation" OR "Developing Nations" OR "Developing Population" OR "Developing Populations" OR "Developing World" OR "LAMI Countries" OR "LAMI Country" OR "Less Developed Countries" OR "Less Developed Country" OR "Less Developed Economies" OR "Less Developed Nation" OR "Less Developed Nations" OR "Less Developed World" OR "Lesser Developed Countries" OR "Lesser Developed Nations" OR lmic OR lmics OR "Low GDP" OR "Low GNP" OR "Low Gross Domestic" OR "Low Gross National" OR "Low Income Countries" OR "Low Income Country" OR "Low Income Economies" OR "Low Income Economy" OR "Low Income Nations" OR "Low Income Population" OR "Low Income Populations" OR "Lower GDP" OR "lower gross domestic" OR "Lower Income Countries" OR "Lower Income Country" OR "Lower Income Nations" OR "Lower Income Population" OR "Lower Income Populations" OR "Middle Income Countries" OR "Middle Income Country" OR "Middle Income Economies" OR "Middle Income Nation" OR "Middle Income Nations" OR "Middle Income Population" OR "Middle Income Populations" OR "Poor Countries" OR "Poor Country" OR " Poor Economies" OR "Poor Economy" OR "Poor Nation" OR "Poor Nations" OR "Poor Population" OR "Poor	

	Populations" OR "poor world" OR "Poorer Countries" OR "Poorer Economies" OR "Poorer Economy" OR "Poorer Nations" OR "Poorer Population" OR " Poorer Populations" OR "Third World" OR "Transitional Countries" OR "Transitional Country" OR "Transitional Economies" OR "Transitional Economy" OR "Under Developed Countries" OR "Under Developed Country" OR "under developed nations" OR "Under Developed World" OR "Under Served Population" OR "Under Served Populations" OR "Underdeveloped Countries" OR "Underdeveloped Country" OR "underdeveloped economies" OR "underdeveloped nations" OR "underdeveloped population" OR "Underdeveloped World" OR "Underserved Countries" OR "Underserved Nations" OR "Underserved Population" OR "Underserved Populations" OR afghanistan OR albania OR algeria OR "American Samoa" OR angola OR argentina OR "Argentine Republic" OR armenia OR azerbaijan OR bangladesh OR belarus OR byelarus OR belorussia OR belize OR benin OR bhutan OR bolivia OR bosnia OR botswana OR brazil OR bulgaria OR burma OR "Burkina Faso" OR burundi OR "Cabo Verde" OR "Cape verde" OR cambodia OR cameroon OR "Central African Republic" OR chad OR china OR colombia OR comoros OR comores OR comoro OR congo OR "Costa Rica" OR "Côte d'Ivoire" OR cuba OR djibouti OR dominica OR "Dominican Republic" OR ecuador OR egypt OR "El Salvador" OR eritrea OR ethiopia OR fiji OR gabon OR gambia OR gaza OR "Georgia Republic" OR georgian OR ghana OR grenada OR grenadines OR guatemala OR guinea OR "Guinea Bissau" OR guyana OR haiti OR herzegovina OR hercegovina OR honduras OR india OR indonesia OR iran OR iraq OR jamaica OR jordan OR kazakhstan OR kenya OR kiribati OR korea OR kosovo OR kyrgyz OR kirghizia OR kirghiz OR kirgizstan OR kyrgyzstan OR "Lao PDR" OR laos OR lebanon OR lesotho OR liberia OR libya OR macedonia OR madagascar OR malawi OR malay OR malaya OR malaysia OR maldives OR mali OR "Marshall Islands" OR mauritania OR mauritius OR mexico OR micronesia OR moldova OR mongolia OR montenegro OR morocco OR mozambique OR myanmar OR namibia OR nauru OR nepal OR nicaragua OR niger OR nigeria OR pakistan OR palau OR panama OR "Papua New Guinea" OR paraguay OR peru OR philippines OR phillippines OR philipines OR phillipines OR principe OR romania OR rwanda OR ruanda OR samoa OR "Sao Tome" OR senegal OR serbia OR "Sierra Leone" OR "Solomon Islands" OR somalia OR "South Africa" OR "South Sudan" OR "Sri Lanka" OR "St Lucia" OR "St Vincent" OR sudan OR surinam OR suriname OR swaziland OR syria OR "Syrian Arab Republic" OR tajikistan OR tadzhikistan OR tadjikistan OR tadzhik OR tanzania OR thailand OR timor OR togo OR tonga OR tunisia OR turkey OR turkmen OR turkmenistan OR tuvalu OR uganda OR ukraine OR uzbek OR uzbekistan OR vanuatu OR venezuela OR vietnam OR "West Bank" OR yemen OR zambia OR zimbabwe)	
5	#1 AND #2 AND #3 AND #4	
6	#5 AND English	939
